# Associations Between Anxiety, Depression, and Disease Activity in Newly Diagnosed Patients with Inflammatory Bowel Disease: A Prospective Cohort Study

**DOI:** 10.3390/nursrep16090301

**Published:** 2026-08-26

**Authors:** Åse Margrete Sagen, Geir Egil Eide, Tore Grimstad, Marit Hegg Reime

**Affiliations:** 1 Department of Internal Medicine, Stavanger University Hospital, Leif Larsens Gate 8, N-4021 Stavanger, Norway; ase.margrete.sagen@sus.no (Å.M.S.); tore.bjorn.grimstad@sus.no (T.G.); 2 Department of Health and Caring Sciences, Faculty of Health and Social Sciences, Western Norway University of Applied Sciences, Inndalsveien 28, N-5020 Bergen, Norway; geir.egil.eide@helse-bergen.no; 3 Department of Clinical Science, University of Bergen, Jonas Lies veg 87, N-5021 Bergen, Norway; 4 Department of Postgraduate Studies, Lovisenberg Diaconal University College, Lovisenberggata 15B, N-0456 Oslo, Norway

**Keywords:** Hospital Anxiety and Depression Scale, inflammatory bowel disease, psychological distress, mental health screening, multivariate regression analyses, nursing

## Abstract

**Background/Objectives**: Psychological distress, including symptoms of anxiety and depression, is common among individuals with inflammatory bowel disease (IBD) and may adversely affect well-being and disease management. However, routine assessment of mental health is not consistently integrated into clinical care. This study assessed the prevalence of anxiety and depression among patients newly diagnosed with IBD at diagnosis and after three months of treatment and explored associations between psychological symptoms, disease activity, and clinical characteristics. **Methods**: This prospective cohort study included 304 adults newly diagnosed with IBD and treated at Stavanger University Hospital between 2012 and 2019. Symptoms of anxiety and depression were assessed using the Hospital Anxiety and Depression Scale at baseline and after three months. Disease activity was evaluated using the Harvey–Bradshaw Index and Partial Mayo Score. Clinical variables included C-reactive protein, faecal calprotectin, folate, cobalamin, and body mass index. Multivariable regression analyses were conducted to identify factors associated with psychological symptoms. **Results**: The prevalence of anxiety decreased from 29.8% at diagnosis to 21.6% after three months, while the prevalence of combined anxiety and depression declined from 22.2% to 16.3%. The prevalence of depression alone remained stable over time. Higher disease activity, particularly as measured by the Harvey–Bradshaw Index, showed the strongest association with anxiety and depressive symptoms. However, after adjustment for potential confounders, no significant improvement in psychological symptoms was observed, suggesting that the association between time and HADS scores may be explained by changes in one or more measured covariates. **Conclusions**: Psychological symptoms remained common during the first months following an IBD diagnosis and were closely associated with disease activity. Medical treatment alone was insufficient to achieve meaningful improvements in mental health outcomes. These findings highlight the importance of systematic psychological assessment, early identification of emotional distress, and integrated multidisciplinary care, including nursing-led screening and supportive interventions, for individuals newly diagnosed with IBD.

## 1. Introduction

Inflammatory bowel disease (IBD), comprising Crohn’s disease (CD) and ulcerative colitis (UC), is a chronic immune-mediated disorder characterized by recurrent inflammation of the gastrointestinal tract [1]. CD can affect any part of the gastrointestinal tract and is often associated with transmural inflammation that often leads to complications such as strictures, fistulae, and intestinal obstruction [1]. UC primarily affects the colonic mucosa in a continuous pattern characterized by alternating periods of remission and relapse, often accompanied by symptoms such as abdominal pain, diarrhea, fatigue, and impaired daily functioning [1]. Both diseases are typically diagnosed during adolescence or early adulthood, a critical period for educational attainment, career development, and relationship formation [2,3]. Experiencing IBD during this formative stage of life may be particularly challenging, as disease symptoms can disrupt social functioning and increase susceptibility to anxiety and depression [4,5]. The unpredictable disease course, fear of flares, and potential surgical interventions may further amplify psychological distress and contribute to concerns about the future [4,5].

The global burden of IBD continues to increase, particularly in Europe and North America, with approximately five million people currently living with the disease worldwide [6]. Norway is among the countries with the highest incidence rates of inflammatory bowel disease, with annual incidences of 14.1–16.0 per 100,000 persons for Crohn’s disease and 24.7–28.4 per 100,000 persons for ulcerative colitis [7]. As no curative treatment currently exists, disease management focuses on symptom control and maintaining remission [8]. Given its chronic and unpredictable nature, IBD has substantial physical, social, and psychological consequences, highlighting the need for comprehensive and person-centred approaches to care [4,9].

Among the most common psychological comorbidities in IBD are symptoms of anxiety and depression [10]. Reported prevalence rates range from 33% to 33.2% for anxiety and from 17.6% to 21.6% for depression [11,12,13]. These symptoms arise through complex interactions between biological and psychosocial factors. Persistent intestinal inflammation, immune dysregulation, alterations in the gut microbiota, and dysregulation of the brain–gut axis have all been suggested as mechanisms contributing to psychological distress in individuals with IBD [14,15]. In addition, the unpredictable disease course, fear of symptom exacerbations, concerns about surgery or long-term treatment, limitations in social participation, and uncertainty regarding the future may contribute to emotional distress and increase vulnerability to anxiety and depressive symptoms [5,16]. Psychological distress is clinically important because it has been linked to impaired quality of life, reduced treatment adherence, difficulties with self-management, increased healthcare utilization, and poorer disease outcomes [9,12].

The close relationship between psychological well-being and IBD has increasingly been understood through the concept of the brain–gut axis, a bidirectional communication network linking the gastrointestinal tract and the central nervous system [17]. Evidence suggests that active disease is associated with an increased risk of anxiety and depression, whereas pre-existing psychological distress may contribute to the onset of IBD [14,15]. Higher levels of stress and negative emotional responses have been associated with poorer clinical outcomes, more frequent disease flare-ups, increased hospitalizations, and greater need for treatment escalation [15,18]. These findings emphasize that psychological symptoms should not be viewed merely as secondary consequences of chronic illness but as factors that may interact with and influence disease activity over time.

Effective coping strategies are important for maintaining psychological well-being among individuals living with IBD [16]. Difficulties in coping with disease-related challenges may contribute to feelings of helplessness and increased anxiety, while negative expectations and diminished hope for the future may heighten susceptibility to depressive symptoms [19]. Consequently, current recommendations advocate routine screening for anxiety and depression and the integration of psychological assessment into comprehensive IBD management [20]. Despite these recommendations, psychological symptoms are not routinely assessed in many clinical settings, and many patients report unmet mental health needs and limited access to psychosocial support services [21,22,23].

Within multidisciplinary IBD care, nurses play a central role in identifying and addressing psychological distress [20]. Through regular contact with patients during outpatient visits, treatment monitoring, patient education, and symptom assessment, nurses are often among the healthcare professionals best positioned to detect early signs of anxiety and depression [24,25]. They contribute to holistic assessment, facilitate communication about emotional concerns, promote adaptive coping strategies, and coordinate referrals to appropriate psychological or psychiatric services when needed [4,5,26]. Patient-reported outcome measures (PROMs), such as the Hospital Anxiety and Depression Scale (HADS), may support this process by providing systematic information about patients’ emotional well-being, enabling more person-centred and individualized care planning [27].

Previous studies have documented the high prevalence of anxiety and depression among individuals with established IBD and have demonstrated associations between psychological distress and adverse clinical outcomes [4,11,12,13]. However, less is known about the prevalence and development of psychological symptoms during the early phase following diagnosis, when patients are adapting to a chronic disease and initiating treatment [28]. Furthermore, there is limited knowledge regarding how anxiety and depression are associated with disease activity shortly after diagnosis and whether these symptoms differ according to gender or IBD subtype during this period [29]. Greater understanding of psychological distress early in the disease trajectory may help clinicians identify patients at risk and support timely, person-centred interventions [30,31]. Therefore, the primary objective was to investigate changes in anxiety and depression during the first three months following treatment initiation in newly diagnosed patients with IBD. Secondary objectives were to identify clinical, biological, and demographic factors associated with psychological distress, thereby informing early assessment and person-centred care strategies.

## 2. Materials and Methods

### 2.1. Design

This hospital-based epidemiological study employed a prospective cohort design and was conducted between 26 April 2012 and 21 January 2019. A prospective observational design was considered appropriate because the primary objective was to examine the occurrence and development of symptoms of anxiety and depression in patients newly diagnosed with IBD during the early phase of standard medical treatment. By following patients from diagnosis onwards without influencing treatment decisions, the study allowed for the assessment of psychological symptoms under routine clinical conditions and for exploration of their relationship with disease activity and clinical characteristics over time.

The study was reported according to the Strengthening the Reporting of Observational Studies in Epidemiology (STROBE) guidelines to ensure transparent and comprehensive reporting of the study design, methods, and findings [32].

### 2.2. Data Collection

Data were collected at baseline and 3 months after initiation of medical treatment as part of the Stavanger University Hospital Inflammatory Bowel Disease (SUSI) study. Participants were recruited using consecutive sampling, resulting in a cohort of 304 newly diagnosed patients with IBD who met the eligibility criteria [33]. The three-month follow-up was selected because this period represents an important early phase of treatment, during which initial therapeutic responses and short-term changes in disease activity are typically observed. Assessing psychological symptoms during this interval provided an opportunity to examine whether anxiety and depression changed during the initial phase of disease management.

Patients aged ≥ 16 years were eligible for inclusion if they had a newly confirmed diagnosis of IBD according to the European Crohn’s and Colitis Organization (ECCO) guidelines [34], had not yet initiated IBD-specific medical treatment, were able to read and understand Norwegian, and were capable of providing written informed consent. Only six participants were aged 16–17 years, reflecting recent changes in the age limits for adult hospital wards in Norway. Exclusion criteria included pregnancy because of potential health risks associated with study participation, as well as cognitive or mental impairments that could limit participants’ ability to comprehend and provide valid responses to the questionnaire.

### 2.3. Instruments and Variables

#### 2.3.1. Psychological Assessment

Symptoms of anxiety and depression were assessed using the HADS [35], a widely used self-report instrument developed for use in clinical populations. The Norwegian version of the HADS has demonstrated satisfactory validity and reliability, with internal consistency values between 0.73 and 0.85 [36]. The instrument consists of 14 items divided into two 7-item subscales measuring anxiety (HADS-A) and depression (HADS-D). Each item is scored on a 4-point Likert scale ranging from 0 to 3, with higher scores indicating greater symptom severity. Subscale scores range from 0 to 21, with scores considered normal (score 0–7), borderline (8–10), or abnormal (≥11) in each domain. Consistent with established recommendations, a score of ≥8 on either the HADS-A or HADS-D subscale was used to indicate clinically relevant symptoms of anxiety or depression, respectively [35]. The total score (HADS-T) ranges from 0 to 42, with a score of ≥15 indicating the presence of concurrent symptoms of anxiety and depression. The HADS was administered at baseline and 3 months after treatment initiation.

#### 2.3.2. Clinical Assessment of Disease Activity

Disease activity in UC was assessed using the Partial Mayo Score (PMS) [37]. The PMS evaluates three domains: stool frequency, rectal bleeding, and the physician’s global assessment of disease activity [37]. Total scores range from 0 to 9, with scores ≤ 2 indicating clinical remission and scores > 7 indicating severe disease activity [38]. Disease activity in CD was assessed using the Harvey–Bradshaw Index (HBI) [39]. The HBI includes measures of general well-being, abdominal pain, number of liquid stools per day, and disease-related complications [39]. HBI scores < 5 were classified as remission, whereas scores > 16 indicated severe disease activity [39].

Both instruments are widely used in clinical practice and research to evaluate disease activity and monitor treatment response in patients with IBD.

#### 2.3.3. Demographic and Clinical Variables

Sociodemographic variables included age and gender. Clinical variables were guided by previous research and clinical relevance and included disease type (UC or CD), biomarkers (C-reactive protein [CRP], folate, cobalamin, faecal calprotectin), disease activity indices (PMS and HBI), and body mass index (BMI). Faecal calprotectin is a widely used biomarker of intestinal inflammation and is useful for distinguishing IBD from functional gastrointestinal disorders [34]. Values > 250 mg/kg were considered indicative of active intestinal inflammation [34]. CRP was included as a marker of systemic inflammation, with elevated levels associated with active disease and clinical relapse in patients with IBD [34]. Folate and cobalamin levels were assessed because deficiencies in these vitamins have been associated with neuropsychiatric symptoms, including anxiety and depression [40]. BMI was also included, as higher BMI has been linked to persistent disease activity and poorer psychological outcomes in individuals with IBD [41]. BMI was calculated as weight in kilograms divided by height in meters squared (kg/m^2^).

### 2.4. Statistical Analysis

The sample size of 304 patients was pragmatically defined based on the available consecutively included IBD population in the study database. Descriptive statistics were used to summarize participant characteristics and study variables. Distribution of continuous variables was presented as means and standard deviations (SDs), or medians and ranges, while categorical variables were presented as frequencies and percentages. Ninety-five percent confidence intervals (95% CIs) were calculated where relevant. Paired comparisons were conducted to examine unadjusted longitudinal changes in anxiety, depression, disease activity, and biomarkers.

Dependent variables included HADS-A, HADS-D, and HADS-T. Normality testing indicated that the HADSs were non-normally distributed; however, given the large sample size, both parametric and non-parametric analyses were performed. Independent samples *t*-tests and Mann–Whitney U tests were used to compare HADS scores across gender and disease type, whereas paired samples *t*-tests and Wilcoxon signed rank tests were used to assess within-subject changes over time. Effect sizes were calculated using Cohen’s *d*. Changes in dichotomized HADS scores were evaluated using McNemar’s test, and Pearson’s correlation coefficient was applied to examine linear associations. Multivariate Analyses of Variance (MANOVA) were used to jointly evaluate multiple dependent variables. Multivariable regression analyses were performed to identify factors associated with anxiety and depression at each assessment point. To study whether the time changes in HADS may work through changes in other potential covariates, mixed linear models (MLMs) were used as the primary longitudinal analyses to evaluate changes in HADS scores over time and account for repeated measurements within individuals, within-subject correlations, and the relevant covariates.

A two-sided *p*-value ≤ 0.05 was considered statistically significant. For multiple comparisons, Bonferroni-adjusted significance levels of *p* ≤ 0.017 were applied [42]. All statistical analyses were conducted using IBM SPSS Statistics version 29.0 (IBM Corp., Armonk, NY, USA).

### 2.5. Ethical Considerations

The SUSI study was approved by the Regional Committee for Medical and Health Research Ethics in 2012 (Reference No. 2011/2631) and conducted in accordance with the ethical principles of the Declaration of Helsinki. All participants received written and oral information about the study and provided written informed consent prior to enrolment. Participation was voluntary, and participants were informed of their right to withdraw from the study at any time without consequences for their treatment or future care.

To protect participant confidentiality, all study data were handled in accordance with applicable data protection regulations and institutional procedures. Personal identifying information was stored separately from research data and replaced with unique study identification numbers. Electronic data were stored on secure, password-protected servers at Stavanger University Hospital, with access restricted to authorized members of the research team. Data were processed and analysed in de-identified form, and all information was treated confidentially throughout the study.

## 3. Results

**Figure 1:**
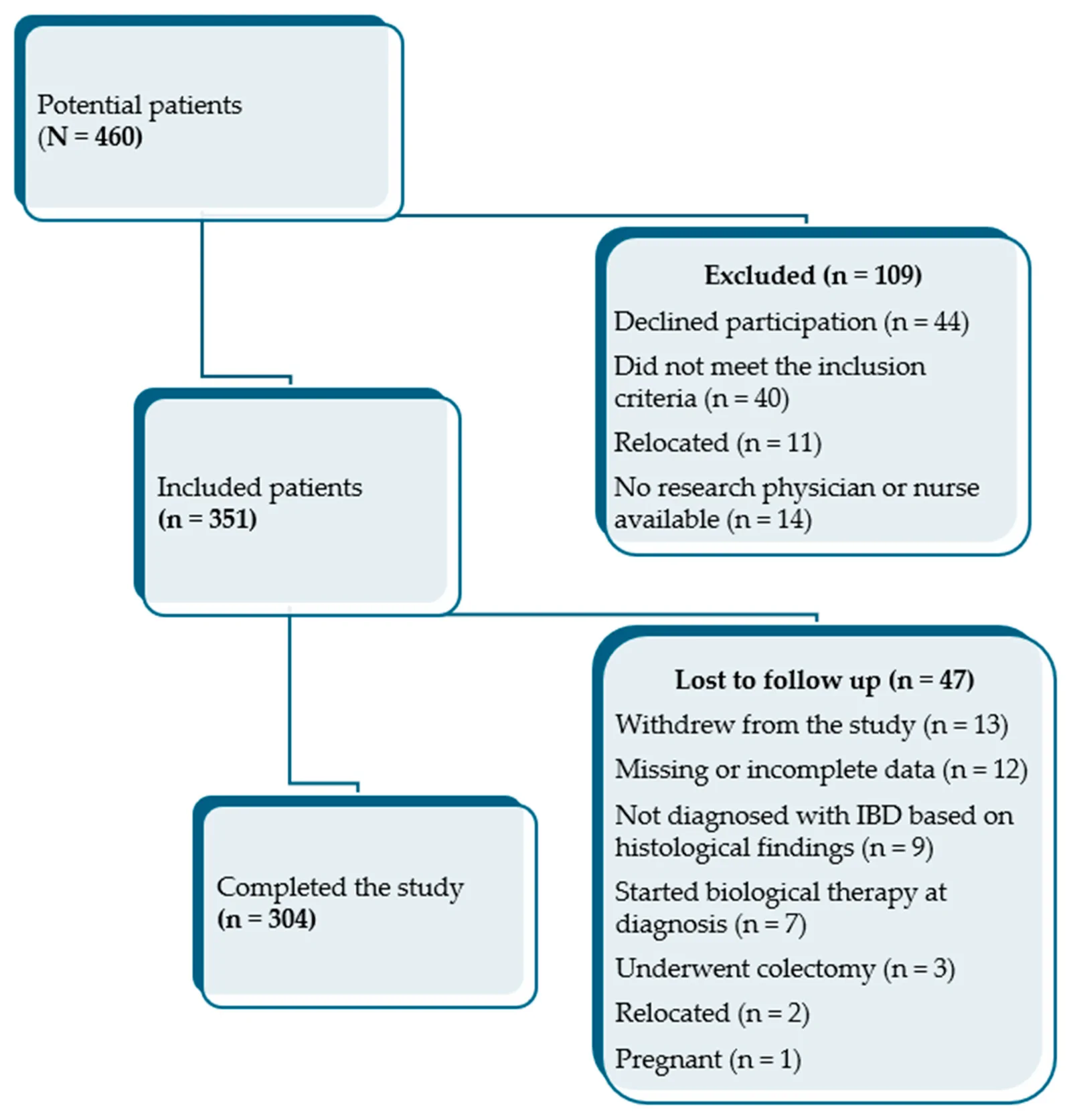
Flowchart illustrating the participants with IBD included and excluded from the Norwegian SUSI Study (2012–2019).

Of the 460 patients assessed for eligibility at Stavanger University Hospital, 109 were excluded due to declined participation (*n* = 44), failure to meet the inclusion criteria (*n* = 40), relocation (*n* = 11), or the unavailability of a research physician or nurse during weekends or holiday periods (*n* = 14). A total of 351 patients were included in the study, of whom 47 were lost to follow-up. Reasons for discontinuation included withdrawal of consent (*n* = 13), missing clinical visits or relevant data (*n* = 12), and the absence of a confirmed IBD diagnosis (*n* = 9). Additionally, 7 patients were deemed ineligible as they initiated biological therapy at diagnosis without prior conventional therapy, 3 underwent colectomy and no longer met the study criteria, 2 relocated, and 1 became pregnant. Consequently, 304 patients completed the study and underwent the final follow-up assessment. The participant recruitment process is illustrated in Figure 1. No significant differences were observed between participants and non-participants with respect to sex distribution or age. Among the 44 individuals who declined participation, 21 (48%) were female, and 23 (52%) were male, with a median age of 34 years, which was comparable to that of the study cohort (Table 1).

### 3.1. Characteristics of the Sample

The baseline characteristics of the study population are presented in Table 1. Among the 304 participants who completed the study, 226 (74.3%) were diagnosed with UC, 164 (53.9%) were men, and the mean age was 37.7 years (range, 16–78 years). The mean BMI was 24.6 kg/m^2^. Most participants (93.1%) received conventional medical therapy in accordance with ECCO guidelines, including one or more of the following treatments: 5-aminosalicylic acid (5-ASA), oral or topical glucocorticoids, or immunomodulators (azathioprine). The remaining 6.9% initiated biological therapy with tumour necrosis factor (TNF) inhibitors, including either infliximab, adalimumab, or certolizumab. At baseline, 29.8% of participants reported symptoms of anxiety, whereas 14.2% reported symptoms of depression based on the predefined HADS cut-off values. Faecal calprotectin measurements were available for 90.5% of the cohort, with a mean concentration of 1111.2 mg/kg, indicating substantial inflammatory activity. The mean CRP level was 19.3 mg/L.

### 3.2. Prevalence of Anxiety and Depression

Significant statistical reductions in psychological distress were observed from baseline to 3 months following treatment initiation (Table 2). Mean scores on all three HADSs—anxiety (HADS-A), depression (HADS-D), and total psychological distress (HADS-T)—decreased significantly over time. Although statistically significant, the magnitude of these changes was small, with Cohen’s *d* effect sizes ranging from −0.19 to −0.26.

When the established HADS cut-off values were applied, the prevalence of anxiety symptoms (HADS-A ≥ 8) decreased significantly from 29.8% at baseline to 21.6% at follow-up. Similarly, the prevalence of combined anxiety and depressive symptoms (HADS-T ≥ 15) decreased significantly from 22.2% to 16.3%. Although the proportion of participants reporting depressive symptoms (HADS-D ≥ 8) decreased from 14.2% to 10.6%, this change did not reach statistical significance (Supplementary Table S1).

### 3.3. Association Between Psychological Symptoms and Disease Activity

HADS-A, HADS-D, and HADS-T scores were significantly associated with disease activity at both baseline and 3-month follow-up. Strong correlations were observed between psychological symptom scores and disease activity indices, including the HBI for Crohn’s disease and the PMS for ulcerative colitis. CRP demonstrated a weak but statistically significant correlation with depressive symptoms (HADS-D). No significant associations were identified between HADS scores and faecal calprotectin, BMI, folate, or cobalamin levels (Supplementary Table S2).

Clinical remission rates increased substantially during the follow-up period. Among patients with UC, the proportion in remission increased from 19.3% at baseline to 78.0% at 3 months. Similarly, remission rates among patients with CD increased from 54.0% to 78.7%. Participants who were in remission at baseline reported significantly lower levels of anxiety and depression compared with those with active disease. At the 3-month follow-up, this association remained significant for anxiety symptoms but not for depressive symptoms (Supplementary Table S3).

### 3.4. Gender and Disease Type Differences

Female participants reported significantly higher anxiety scores (HADS-A) than male participants at both baseline and the 3-month follow-up. Women also had significantly higher total HADS scores (HADS-T) at 3 months; however, the observed effect sizes were small, suggesting limited clinical relevance. No significant sex differences were found for depressive symptoms (HADS-D) at either assessment point (Supplementary Table S4).

No significant differences in HADS-A, HADS-D, or HADS-T scores were observed between patients with UC and those with CD at baseline or at the 3-month follow-up (Supplementary Table S5). However, when changes over time were examined within each diagnostic group, patients with UC demonstrated significant reductions in both anxiety and depressive symptoms, whereas HADS scores remained largely unchanged among patients with CD (Supplementary Table S6).

### 3.5. Associated Factors

Multivariable regression analyses identified higher disease activity, reflected by higher HBI and PMSs, and younger age as the strongest correlates of greater psychological distress at baseline, as indicated by higher HADS-A, HADS-D, and HADS-T scores. At the 3-month follow-up, higher HBI scores and younger age remained significantly associated with psychological distress, whereas PMS was no longer significantly associated with psychological outcomes. Neither inflammatory biomarkers (faecal calprotectin and CRP), nutritional markers (cobalamin and folate), nor BMI were significantly associated with HADS scores at baseline or at the 3-month follow-up.

Among the examined variables, HBI emerged as the strongest correlate of psychological symptoms, explaining 7.3% of the variance in HADS-A (Adjusted R^2^ = 0.073), 12.8% in HADS-D (Adjusted R^2^ = 0.128), and 14.1% in HADS-T (Adjusted R^2^ = 0.141) scores (Table 3). The association between HBI and psychological symptoms remained evident at the 3-month follow-up, while the association with age attenuated over time (Supplementary Table S7). Longitudinal analyses using mixed linear models showed no significant changes in HADS-A, HADS-D, or HADS-T scores between baseline and 3 months after adjustment for relevant covariates. These findings were consistent regardless of whether disease activity was represented by HBI or PMS in the models (Supplementary Table S8).

## 4. Discussion

This study examined changes in symptoms of anxiety and depression during the first 3 months after initiation of medical treatment in patients newly diagnosed with IBD and explored their associations with clinical, biological, and demographic factors. By focusing on the early post-diagnostic phase, a period that has received comparatively little attention, this study adds to the existing literature on psychological symptoms in IBD. Whereas previous studies and meta-analyses have largely focused on the prevalence of psychological distress in heterogeneous cohorts with varying disease durations [12,13,29], the present study provides insight into psychological symptoms at diagnosis and during the first three months following treatment initiation. This early phase is clinically important as patients are simultaneously adapting to a chronic illness, initiating treatment, and developing strategies for long-term disease management. A novel finding of this study is that subjective measures of disease activity were more strongly associated with psychological distress than objective biomarkers. This observation underscores the importance of understanding patients’ lived experiences of the disease, rather than relying solely on biological markers of inflammation.

At baseline, 29.8% of participants reported clinically relevant anxiety symptoms and 14.2% reported depressive symptoms. These findings are consistent with previous studies demonstrating that individuals with IBD experience higher levels of psychological distress than the general population, particularly during the period surrounding diagnosis and treatment initiation [11,12,13]. After three months of treatment, unadjusted paired analyses showed significant reductions across all HADS scores. However, these are crude differences that should be interpreted with caution. Following adjustment for relevant covariates including disease activity and demographic variables in mixed linear regression models, no statistically significant reductions were observed in anxiety or depression symptoms over time. These findings suggest that initiation of medical treatment alone may have a limited short-term impact on psychological well-being in patients with IBD. Alternatively, the three-month follow-up period may have been too brief to capture meaningful improvements, particularly for depressive symptoms, which often develop and resolve more gradually than disease-related physical symptoms [43]. Although mean HADS-A and HADS-D scores decreased over time, the observed reductions (0.75 and 0.57 points, respectively) were below the established minimal clinically important difference (MCID) of 1.7 points [44], suggesting that these changes were unlikely to be perceived as clinically meaningful by patients. This interpretation is further supported by the small effect sizes observed for anxiety (Cohen’s *d* = −0.26) and depression (Cohen’s *d* = −0.19), indicating that the overall magnitude of improvement was modest. Together, these findings underscore the important distinction between statistical significance and clinical significance.

While anxiety and depressive symptoms improved following diagnosis and treatment initiation, the extent of change may have been insufficient to translate into meaningful improvements in patients’ psychological well-being. Consequently, many individuals are likely to continue experiencing clinically relevant psychological distress despite improvements in disease-related outcomes. These findings highlight the persistent psychological burden associated with IBD during the early phase of treatment and suggest that improvements in disease activity alone may not be sufficient to promote meaningful psychological recovery [29]. From a nursing perspective, this underscores the importance of routinely assessing psychological distress and integrating psychosocial support alongside medical management from an early stage of care.

The relative stability of anxiety and depressive symptoms following treatment initiation suggests that monitoring disease activity alone may be insufficient to identify patients experiencing psychological distress. IBD nurses play a key role in patient education, symptom monitoring, follow-up care, and the identification of psychosocial concerns and are therefore well positioned to initiate discussions about psychological wellbeing and facilitate referral when additional support is needed [20]. Incorporating psychological assessment into routine care, such as HADS into routine nursing assessments, may facilitate timely recognition of patients who require additional support. This is also consistent with principles of person-centred care, which emphasize addressing patients’ emotional and psychosocial needs alongside physical health concerns [45]. Taken together, these findings support integrating psychological assessment early in the disease trajectory rather than reserving such evaluations for patients with longstanding disease.

Unexpectedly, biomarkers commonly used to assess disease activity, including faecal calprotectin, CRP, cobalamin, folate, and BMI, were not associated with anxiety or depression symptoms as measured by the HADS at either assessment point. Although the brain–gut axis provides an important framework for understanding interactions between inflammation and psychological well-being [14,28], these findings suggest that routinely used biomarkers may not adequately reflect patients’ psychological experiences. This observation is consistent with previous research demonstrating that emotional well-being is more strongly associated with symptom burden, fatigue, and illness perceptions than with biochemical indicators of disease activity alone. In line with these findings, symptom-based measures, including the Harvey-Bradshaw Index and the Partial Mayo Score, were consistently associated with symptoms of anxiety and depression. The Harvey–Bradshaw Index showed the strongest association with psychological distress, likely because it incorporates patient-reported experiences such as pain, fatigue, and overall well-being, which are closely linked to mental health [26,46]. This interpretation is supported by Azizoglu, Kurt, and Tezel (2025) [47], who found that disease activity, rather than disease subtype (UC/CD), was the strongest correlate of psychological morbidity in patients with IBD, with one in three patients meeting clinical depression thresholds. All these findings highlight the importance of person-centred nursing assessments that extend beyond objective disease markers and include patients’ subjective symptom experiences. From a nursing perspective, the results highlight the importance of person-centred assessments that extend beyond objective disease markers to include symptom burden and psychological well-being. Comprehensive care for individuals with IBD should therefore integrate evaluation of both physical and emotional health.

Gender differences were evident in the present study, with women reporting significantly higher anxiety scores than men at both assessment points. This finding is consistent with previous research demonstrating greater susceptibility to anxiety symptoms among women with IBD and other chronic conditions [12,47,48,49,50]. In contrast, no significant gender differences were observed in depression scores. These results suggest that gender may be an important consideration when assessing psychological well-being in individuals with IBD.

No significant differences in anxiety or depression were found between patients with ulcerative colitis and Crohn’s disease. This contrasts with some previous studies that have reported a greater psychological burden among patients with Crohn’s disease, potentially related to the more complex and progressive nature of the disease [12,26,51]. Our findings indicate that psychological distress may not be determined by disease subtype alone and support the need to assess mental health concerns across the entire IBD population.

Age also emerged as a relevant factor, particularly among patients with UC, with younger individuals reporting higher anxiety levels. This observation is in line with previous research suggesting that younger adults with chronic illnesses are at increased risk of psychological distress [48]. Factors such as uncertainty about long-term disease management, disruptions to education and employment, and challenges related to social and family life may contribute to heightened anxiety in this group [47]. Together, these findings highlight the importance of incorporating routine assessment of psychological well-being into person-centred IBD care, with particular attention to younger patients and women who may be more vulnerable to anxiety symptoms. Although the HBI and PMS were significantly associated with psychological distress, the relatively modest proportion of explained variance suggests that psychological distress in IBD is influenced by factors beyond disease activity alone. Indeed, psychological distress in IBD is increasingly understood within a biopsychosocial framework, in which biological factors (e.g., intestinal inflammation and disease activity), psychological factors (e.g., stress, coping, and illness perceptions), and social factors (e.g., social support and life circumstances) interact to influence anxiety and depressive symptoms [52]. Consistent with this framework, previous research has identified psychosocial and behavioural factors, including coping strategies, fatigue, body image concerns, social support, and lifestyle behaviours as important determinants of psychological well-being [19,26,53,54,55]. The multifactorial nature of psychological distress in IBD highlights the need for a holistic approach that considers both clinical and psychosocial dimensions of health. Future research should incorporate these factors to improve understanding of psychological vulnerability in IBD and support the development of more targeted, person-centred interventions aimed at promoting psychological well-being and overall quality of life.

The high prevalence of anxiety and depression among individuals with IBD, combined with the limited improvement observed over the three-month follow-up period, highlights the persistent psychological burden associated with the disease. These findings support the importance of addressing psychological well-being as an integral component of IBD care. Routine assessment of anxiety and depression may facilitate the early identification of patients experiencing psychological distress and may help ensure that patients with persistent or severe symptoms receive appropriate care [56]. Psychological interventions, including cognitive behavioural therapy, stress-management programmes, and coping-skills training, have shown potential for improving mental well-being and quality of life among individuals with chronic diseases [56]. Such interventions may [19] help patients develop adaptive coping strategies and improve their ability to manage the challenges associated with living with a chronic condition [29]. Although selected psychological interventions have shown beneficial effects on psychological wellbeing in chronic illness populations, the present study did not evaluate specific interventions and, therefore, no conclusions can be drawn regarding treatment efficacy in this cohort.

The persistence of anxiety and depression during the first months following diagnosis suggests that psychological wellbeing should be addressed alongside symptom control from the outset of care. Incorporating routine patient-reported outcome measures, such as the HADS, may help nurses and clinicians identify individuals requiring additional support and facilitate more personalized care planning. The findings align with principles of person-centred care, which emphasize the importance of understanding patients’ lived experiences, individual preferences, and psychosocial circumstances alongside biological indicators of disease [57]. Such an approach highlights the need for multidisciplinary care, where gastroenterologists, IBD nurses, primary care providers, and mental health professionals work collaboratively to address the complex and interconnected needs of individuals living with IBD.

### Strengths and Limitations

A particular strength and novel contribution of this study is the focus on the early post-diagnostic period in a relatively large cohort of newly diagnosed patients with IBD. There were no power calculations done beforehand, and post hoc power calculation was futile [58]. We have, however, done some estimates for a potential new study. In a new study to detect an effect given by Cohen’s *d* = 0.20 (‘small effect’), a study of the same size will have a power of 93%. Correspondingly, to achieve a statistical power of 80% (90%), a sample size of 199 (265) participants would be required.

The prospective cohort design and large sample size (*n* = 304) enhance the reliability of the findings and support their applicability to a clinical IBD population. The use of validated and widely used instruments, including the Hospital Anxiety and Depression Scale, the Harvey–Bradshaw Index, and the Partial Mayo Score, strengthens methodological rigor and facilitates comparison with previous studies. Furthermore, application of Bonferroni correction reduced the risk of type I error associated with multiple statistical comparisons, thereby increasing confidence in the reported findings. Another strength is the inclusion of both clinical disease activity measures and biological markers alongside psychological assessments, providing a more comprehensive understanding of factors associated with anxiety and depression in individuals with IBD. This multidimensional approach contributes to the growing evidence that psychological well-being in IBD cannot be fully understood through biological indicators alone and underscores the importance of assessing both physical and psychosocial aspects of health.

Several limitations should be considered when interpreting the findings of this study. First, the observational design precludes causal inferences, limiting conclusions regarding whether changes in psychological symptoms were directly related to treatment effects or influenced by other unmeasured factors.

Second, the study was conducted at a single centre, which may limit the generalizability of the findings due to selection bias, as the study population may not be fully representative of patients with IBD treated in other healthcare settings or geographical regions. Differences in patient characteristics, disease management practices, and healthcare delivery may influence the applicability of the results to other IBD populations.

Third, the three-month follow-up period may have been insufficient to capture clinically meaningful changes in psychological outcomes, particularly depression, which may require longer periods to respond to treatment and adaptation processes. In addition, the use of self-reported measures may have introduced reporting bias, including the potential under- or overestimation of psychological symptoms. Although validated instruments were employed, self-reported assessments remain subject to individual interpretation and response tendencies. By inviting all patients with IBD treated at our centre to participate, we aimed to reduce selection bias. However, participant exclusions and loss to follow-up may have introduced attrition bias if individuals who did not complete the 3-month assessment differed systematically from those who remained in the study.

In addition, volunteer bias is an inherent limitation of observational research [59], and it is possible that patients with symptoms of anxiety or depression were more motivated to participate, potentially resulting in an overestimation of psychological distress within the cohort.

In the analyses with multivariate mixed linear regression models, there was no consistent significant regression coefficient for time (Supplementary Table S8). This may be different using a causal modelling approach, but not having such a model prespecified, we do not think it is appropriate to do so within this observational study design. In the light of post hoc reflection, it may be that adjusting for the covariates that are measured on both occasions may distort the time effect on HADS as time works through the covariates on HADS.

Furthermore, the cohort was recruited between 2012 and 2019, and changes in IBD management over time may limit the direct applicability of the findings to current clinical practice. Although the relationship between disease activity and psychological distress is likely to remain relevant, shifts in therapeutic approaches and standards of care may influence the magnitude and clinical expression of these associations in contemporary patient populations.

Finally, despite adjustment for several relevant demographic and clinical variables, residual confounding remains possible. Important psychosocial, sociodemographic, and disease-related factors that may influence both disease activity and psychological distress were not assessed. These factors include coping strategies, fatigue, social support, illness perceptions, life stressors, educational level, marital or cohabitation status, employment status, smoking status, bowel urgency, sleep disturbances, fear of relapse, perceived social stigma, comorbidities, surgical history, disease location and phenotype, perianal disease, and hospitalization history. In addition, information on previous psychiatric history, use of antidepressants or anxiolytics, and access to psychological or psychiatric care during the study period, including psychological support and IBD nurse follow-up, was unavailable. These unmeasured factors may have influenced psychological well-being and may partly explain the relatively modest proportion of variance in anxiety and depression accounted for by the variables included in the analyses. Future studies should incorporate a broader range of demographic, clinical, and biological variables, as well as longer follow-up periods, to provide a more comprehensive understanding of psychological distress in individuals with IBD.

Future studies should incorporate a broader range of demographic, clinical, and biological variables, as well as longer follow-up periods, to better elucidate the factors associated with psychological distress in individuals with IBD. Psychological adaptation to an IBD diagnosis may unfold over a considerably longer timeframe than captured in the present study; thus, longer follow-up periods may offer a more nuanced understanding of the trajectory of anxiety and depressive symptoms after diagnosis and treatment initiation. Such follow-up is particularly relevant because depression may evolve more gradually than disease activity, while anxiety associated with a new diagnosis may fluctuate considerably during the early phases of treatment [28,29]. This notion is supported by longitudinal evidence demonstrating that only 10% of patients reporting anxiety or depressive symptoms at baseline experienced improvement over time, suggesting that psychological distress often persists despite ongoing disease management. Accordingly, extended follow-up may be necessary to capture longer-term patterns of psychological adaptation and recovery in individuals with IBD [28].

## 5. Conclusions

This study provides evidence that symptoms of anxiety and depression are highly prevalent at the time of IBD diagnosis and may persist despite initial medical treatment. Symptom-based disease activity, rather than objective inflammatory biomarkers, was most closely associated with psychological distress, underscoring the importance of considering patients’ subjective experiences alongside clinical indicators. The findings support early psychological assessment as part of person-centred IBD care and highlight the need for future research exploring biopsychosocial factors that contribute to psychological well-being following diagnosis.

## Figures and Tables

**Table 1:** Baseline Characteristics of 304 Patients with IBD Included in the Norwegian SUSI Study (2012–2019).

Variables	Groups	*n*	Range	Md	Mean	SD	%	95% CI
Age, years		304	16–78	34	37.7	15.4		(36.0, 39.4)
Gender	Male	164					53.9	(46.3, 61.5)
	Female	140					46.1	(37.8, 54.4)
Diagnosis	UC	226					74.3	(68.6, 80.0)
	CD	78					15.7	(7.6, 23.8)
HADS-A (scale 0–21)		302	0–19	5	5.76	3.9		(5.32, 6.20)
HADS-D (scale 0–21)		302	0–17	3	3.43	3.4		(3.05, 3.81)
HADS-T (scale 0–42)		302	0–35	8	9.1	6.5		(8.37, 9.83)
Anxiety (HADS-A ≥ 8)	yes	90					29.8	(20.4, 39.2)
	no	212					70.2	(64.0, 76.4)
Depression (HADS-D ≥ 8)	yes	43					14.2	(3.8, 24.6)
	no	259					85.8	(81.5, 90.1)
Combined (HADS-T ≥ 15)	yes	67					22.2	(12.2, 32.2)
	no	235					77.8	(72.5, 83.1)
Treatment	Biological ^(a)^	21					6.9	(0, 17.7)
	Conventional ^(b)^	283					93.1	(90.1, 96.1)
	5-ASA	226					74.3	(68.6, 80.0)
	Glucocorticoids	138					45.4	(37.1, 53.7)
	Azathioprine	18					5.9	(0, 16.8)
Harvey Bradshaw Index (scale: 0–50)		79	0–16	5	5.3	3.6		(4.5, 6.1)
Partial Mayo Score (scale: 0–9)		223	0–9	5	4.98	2.43		(4.66, 5.30)
Faecal Calprotectin, mg/kg		275	15–6000	668	1111.2	1122.2		(978.6, 1243.8)
C-Reactive Protein, mg/L		303	1–184	6.3	19.3	30.7		(15.8, 22.8)
Folate, nmol/L		297	1.2–63	12	14.1	8		(13.2, 15.0)
Cobalamin, pmol/L		303	114–1400	370	424.8	235.5		(398.3, 451.3)
Body Mass Index (BMI), kg/m^2^		297	14.2–47.9	24.3	24.6	4.6		(24.1, 25.1)
Underweight (<18.5)		18					6.1	(0, 17.2)
Normal weight (18.5–24.9)		154					51.8	(43.9, 59.7)
Overweight (25.0–29.9)		93					31.3	(21.9, 40.7)
Obese (≥30)		32					10.8	(0.0, 21.6)

*Abbreviations*: SUSI = Stavanger University Hospital IBD Study; IBD = Inflammatory Bowel Disease; HADS = Hospital Anxiety and Depression Scale; HADS-A = Anxiety Subscale; HADS-D = Depression Subscale; Min = Minimum Observed Value; Max = Maximum Observed Value; SD = Standard Deviation; Md = Median, CI = confidence interval (normal approximated. Negative lower limits are set to 0); UC = Ulcerative Colitis; CD = Crohn’s Disease. Reference Ranges: CRP (<5); Folate (>6.8); Cobalamin cutoff for disease indication (>250); BMI (18.5–24.9); Calprotectin: Normal (<50); Cutoff for disease indication (>250). ^(a)^ Biological Treatment = Infliximab, Adalimumab, Certolizumab; ^(b)^ Conventional Treatment = 5 ASA (5-aminosalicylic acid) and/or glucocorticoids (administered orally and/or topically), and/or immunosuppressives (Azathioprine).

**Table 2:** Changes in HADS Scores from Baseline to Three Months Post-Treatment for 304 Patients with IBD Included in the Norwegian SUSI Study (2012–2019).

	Time	Descriptives			Change from 0 to 3 Months					
Variables	m	Mean	SD	Md	Mean	SD	95% CI	*t*-Test *p*	W *p*	d
HADS-A	0	5.76	3.90	5						
	3	5.02	3.67	4	−0.75	2.91	(−1.08, −0.41)	**0.001**	**<0.001**	−0.26
HADS-D	0	3.43	3.39	3						
	3	2.87	3.23	2	−0.57	3.03	(−0.92, −0.23)	**<0.001**	**0.007**	−0.19
HADS-T	0	9.19	6.60	8						
	3	7.89	6.33	6	−1.32	5.15	(−1.90, −0.73)	**<0.001**	**<0.001**	−0.26

Abbreviations: SUSI = Stavanger University Hospital IBD Study; IBD = Inflammatory Bowel Disease; HADS = Hospital Anxiety and Depression Scale; HADS-A = Anxiety Subscale; HADS-D = Depression Subscale; HADS-T = Combined Anxiety and Depression Subscale; m = months; Md = Median SD = Standard Deviation; CI = Confidence Interval; *t*-test = Paired *t*-test; W = Wilcoxon test; d = Cohen’s *d.* (**Bold** = *p* ≤ 0.05).

**Table 3:** Joint Linear Regressions of HADS-A, HADS-D, and HADS-T at Baseline and Three Months Post-Treatment on Diagnosis (UC/CD), HBI, and PMS Models ^(a)^, Adjusted for Other Covariates, in 304 Patients with IBD Included in the Norwegian SUSI Study (2012–2019).

Covariates	Diagnosis Models		HBI Models		PMS Models	
0 MONTHS	Wilks’ Lambda	*p*	Wilks’ Lambda	*p*	Wilks’ Lambda	*p*
Intercept	0.90	<0.001	0.90	0.038	0.94	0.005
Diagnosis (UC)	1.00	0.764				
HBI (scale: 0–50)			0.86	**0.010 ^(b)^**		
PMS (scale: 0–9)					0.95	**0.015 ^(b)^**
CRP, mg/L	0.97	0.037	1.00	0.906	0.97	0.040
Calprotectin, mg/kg	0.99	0.308	0.97	0.345	0.98	0.143
Folate, nmol/L	0.99	0.372	0.99	0.783	0.99	0.283
Cobalamin, pmol/L	1.00	0.917	0.98	0.509	1.00	0.953
BMI, kg/m^2^	1.00	0.780	0.99	0.680	1.00	0.856
Age, years	0.95	**0.003 ^(b)^**	0.99	0.816	0.94	**0.005 ^(b)^**
Gender (Male)	0.99	0.526	0.94	0.168	0.99	0.416
Adjusted R^2^	(*n* = 261)		(*n* = 69)		(*n* = 191)	
HADS-A	0.032		0.061		0.088	
HADS-D	0.002		0.045		0.021	
HADS-T	0.0013		0.068		0.058	
**3 MONTHS**	**Wilks’ Lambda**	***p***	**Wilks’ Lambda**	***p***	**Wilks’ Lambda**	***p***
Intercept	0.92	<0.001	0.96	0.320	0.89	<0.001
Diagnosis (UC)	0.99	0.324				
HBI (scale: 0–50)			0.82	**0.005 ^(b)^**		
PMS (scale: 0–9)					0.96	0.042
CRP, mg/L	0.99	0.277	0.98	0.646	0.98	0.119
Calprotectin, mg/kg	1.00	0.799	0.99	0.734	1.00	0.846
Folate, nmol/L	0.99	0.316	0.99	0.697	0.98	0.220
Cobalamin, pmol/L	0.99	0.269	0.95	0.296	0.99	0.471
BMI, kg/m^2^	0.98	0.106	0.98	0.646	0.98	0.119
Age, years	0.97	0.036	0.99	0.866	0.95	**0.017 ^(b)^**
Gender (male)	0.99	0.235	0.98	0.607	0.98	0.258
Adjusted R^2^	(*n* = 238)		(*n* = 62)		(*n* = 176)	
HADS-A	0.014		0.073		0.030	
HADS-D	0.013		0.128		0.025	
HADS-T	0.006		0.141		0.017	

Note: Wilks’ Lambda is a statistical test utilized to evaluate significant differences between groups across a combination of dependent variables. Lower values suggest greater differences between groups. Abbreviations: SUSI = Stavanger University Hospital IBD Study; IBD = Inflammatory Bowel Disease; HADS = Hospital Anxiety and Depression Scale; HADS-A = Anxiety Subscale; HADS-D = Depression Subscale; HADS-T = Combined Anxiety and Depression Subscale; Diagnosis = Ulcerative Colitis vs. Crohn’s Disease; HBI = Harvey-Bradshaw Index; PMS = Partial Mayo Score; CRP = C-Reactive Protein; BMI = Body Mass Index. Dependent Variables: HADS-A, HADS-D, HADS-T. Reference Ranges: CRP (<5); Folate (>6.8); Cobalamin cutoff for disease indication (>250); BMI (18.5–24.9); Calprotectin: Normal (<50); Cutoff for disease indication (>250). ^(a)^ Six models were developed based on two time points (0 and 3 months) and three covariates (Diagnosis, HBI, and PMS), with each covariate included one at a time to address issues of missing values, reduce multicollinearity, and enhance the interpretation of covariate effects. ^(b)^ **Bold**; *p*-values were deemed significant at Bonferroni-corrected alpha = 0.05/3 (for three dependent variables) = 0.017.

## Data Availability

All relevant data generated or analysed during this study are included in this published article and its Supplementary Materials.

## Supplementary Materials

The following supporting information can be downloaded at https://www.mdpi.com/article/10.3390/nursrep16090301/s1. Table S1: Changes in Dichotomized HADS Scores; Table S2: Correlation Between HADS Scores and Disease Indicators, Biomarkers, and Body Mass Index; Table S3: Prevalence and Impact of Clinical Remission on Anxiety and Depression; Table S4: Comparison of HADS Scores Between Males and Females; Table S5: Comparison of HADS Scores Between Patients with UC and CD; Table S6: Differences between UC and CD in Dichotomized HADS Prevalence Scores; Table S7: Results from linear regression analyses; Table S8: Results from mixed linear regression models.

## Public Involvement Statement

Patients and members of the public were not involved in the design, conduct, reporting, or dissemination plans of this research.

## Guidelines and Standards Statement

This manuscript was prepared in accordance with the Strengthening the Reporting of Observational Studies in Epidemiology (STROBE) guidelines for cohort studies.

## Abbreviations

The following abbreviations are used in this manuscript:

IBD	Inflammatory Bowel Disease
HADS	Hospital Anxiety and Depression Scale
HADS-A	Hospital Anxiety and Depression Scale-Anxiety subscale
HADS-D	Hospital Anxiety and Depression Scale-Depression subscale
HADS-T	Hospital Anxiety and Depression Scale-Total score
UC	Ulcerative Colitis
CD	Crohn’s Disease
HBI	Harvey–Bradshaw Index
PMS	Partial Mayo Score
CRP	C-reactive protein
BMI	Body mass index
