# Supplementary material for: Associations Between Anxiety, Depression, and Disease Activity in Newly Diagnosed Patients with Inflammatory Bowel Disease: A Prospective Cohort Study
Source: Nurs Rep. 2026 Aug 26;16(9):301. doi: 10.3390/nursrep16090301 (PMC13610852; doi:10.3390/nursrep16090301)
Supplement: Supplementary file 1 [file nursrep-16-00301-s001.zip › Supplementary Table S8.pdf]

Supplementary Table S8. Mixed linear regression models of HADS-A, HADS-D, and HADS-T, assessed jointly at baseline and 3 months post-treatment, based on diagnosis (UC/CD), HBI, and PMS models a), adjusted for other potential predictor variables, for n = 304 patients with IBD included in the Norwegian SUSI study (2012-2019).

**Predictors**	**Model 1 with diagnosis**		**Model 2 with HBI**		**Model 3 with PMS**	
**HADS-A**	**b (coefficient)**	**P-value**	**b (coefficient)**	**P-value**	**b (coefficient)**	**P-value**
Intercept	6.99	< 0.001	3.98	0.078	6.38	< 0.001
Diagnosis (UC)	-0.19	0.697				
HBI (scale: 0-50)			0.34	**< 0.001[b)]**		
PMS (scale: 0-9)					0.22	**0.010[b)]**
CRP, *mg/L*	0.00	0.548	0.01	0.651	0.00	0.571
Calprotectin, *mg/kg*	0.00	0.504	0.00	0.098	0.00	0.276
Folate, *nmol/L*	0.03	0.131	0.05	0.080	0.02	0.500
Cobalamin, *pmol/L*	0.00	0.553	0.00	0.257	0.00	0.409
BMI, *kg/m²*	0.01	0.856	0.05	0.583	0.01	0.900
Age, *years*	-0.04	**0.005[b)]**	-0.02	0.415	-0.05	**0.006[b)]**
Male	-0.88	0.036	0.19	0.793	-1.01	0.042
Time (3m/0m)	-0.64	**0.008[b)]**	-0.23	0.594	-0.07	0.854
**HADS-D**	**B**	**P-value**	**B**	**P-value**	**B**	**P-value**
Intercept	2.48	0.027	0.24	0.912	1.09	0.420
Diagnosis (UC)	0.57	0.178				
HBI (scale: 0-50)			0.36	**< 0.001[b)]**		
PMS (scale: 0-9)					0.24	**0.003[b)]**
CRP, *mg/L*	0.01	0.038	0.00	0.951	0.01	0.355
Calprotectin, *mg/kg*	0.00	0.253	0.00	0.282	0.00	0.735
Folate, *nmol/L*	-0.01	0.595	0.02	0.516	-0.03	0.273
Cobalamin, *pmol/L*	0.00	0.459	0.00	0.564	0.00	0.337
BMI, *kg/m²*	0.05	0.225	0.07	0.419	0.06	0.232
Age, *years*	-0.01	0.484	-0.01	0.844	-0.01	0.506
Male	-0.33	0.370	0.92	0.192	-0.57	0.174
Time (3m/0m)	-0.16	0.501	0.08	0.841	0.54	0.134
**HADS-T**	**B**	**P-value**	**B**	**P-value**	**B**	**P-value**
Intercept	9.63	< 0.001	4.03	0.294	7.65	0.004
Diagnosis (UC)	-0.78	0.350				
HBI (scale: 0-50)			0.68	**< 0.001[b)]**		
PMS (scale: 0-9)					0.45	**0.002[b)]**
CRP, *mg/L*	0.01	0.158	0.01	0.718	0.00	0.922
Calprotectin, *mg/kg*	0.00	0.798	0.00	0.112	0.00	0.648
Folate, *nmol/L*	0.02	0.627	0.07	0.143	-0.01	0.758
Cobalamin, *pmol/L*	0.00	0.407	0.00	0.353	0.00	0.276
BMI, *kg/m²*	0.06	0.514	0.13	0.408	0.06	0.530
Age, *years*	-0.05	0.050	-0.03	0.527	-0.06	0.050
Male	-1.22	0.091	1.08	0.395	-1.60	0.058
Time (3m/0m)	-0.80	0.056	-0.18	0.797	0.46	0.480

Abbreviations: *SUSI* = Stavanger University Hospital IBD study; *IBD* = Inflammatory Bowel Disease; *HADS* = Hospital Anxiety and Depression Scale; *HADS-A* = Anxiety subscale; *HADS-D* = Depression subscale; *HADS-T* = Combined anxiety and depression subscale; *Diagnosis* = Ulcerative colitis (UC) versus Crohn's disease (CD); *HBI* = Harvey-Bradshaw Index; *PMS* = Partial Mayo Score; *CRP* = C-Reactive Protein; *BMI* = Body Mass Index. **Reference Ranges:** *CRP* (<5); *Folate* (>6.8); *Cobalamin cutoff for disease indication* (>250); *BMI* (18.5-24.9); *Calprotectin*: Normal (<50); Cutoff for disease indication (>250); **Dependent**

Variables: *HADS-A*, *HADS-D*, *HADS-T*. a) Nine models were developed based on three outcomes (HADS-A, HADS-D, HADS-T) and three predictors (Diagnosis, HBI, and PMS), with each predictor included one at a time to address issues related to missing values when included simultaneously, reduce the risk of multicollinearity, and facilitate easier interpretation of the predictor effects. b) **Bold**; P-values were considered significant at Bonferroni-corrected alpha = 0.05/3 (for three dependent variables) = 0.017.
