# Supplementary material for: Associations Between Anxiety, Depression, and Disease Activity in Newly Diagnosed Patients with Inflammatory Bowel Disease: A Prospective Cohort Study
Source: Nurs Rep. 2026 Aug 26;16(9):301. doi: 10.3390/nursrep16090301 (PMC13610852; doi:10.3390/nursrep16090301)
Supplement: Supplementary file 1 [file nursrep-16-00301-s001.zip › Supplementary Table S7.pdf]

Supplementary Table S7. Results from linear regression analyses of HADS-A, HADS-D and HADS-T on HBI, PMS and UC/CD-diagnosis, respectively, at 0 and 3 months adjusted for other potential confounders[a)] in 304 patients included in the Norwegian SUSI study (2012-2019)[b)].

HADS outcome	Significant predictors	0 months B	p-value	3 months B	p-value
	**Harvey Bradshaw index** (scale: 0-25)				
**HADS-A**		0.43	**0.004** [c)]	0.54	**0.003** [c)]
**HADS-D**		0.38	**0.009** [c)]	0.46	**0.006** [c)]
**HADS-T**		0.80	**0.002** [c)]	1.00	**0.001** [c)]
**HADS-A**	Age in years	-0.01	0.700	-0.02	0.635
**HADS-D**		0.01	0.874	-0.01	0.645
**HADS-T**		-0.01	0.899	-0.03	0.590
	**Partial Mayo Score** (scale: 0-9)				
**HADS-A**		0.36	**0.005** [c)]	0.19	0.319
**HADS-D**		0.13	0.252	0.38	0.020
**HADS-T**		0.49	0.024	0.57	0.083
**HADS-A**	Age in years	-0.06	**0.002** [c)]	-0.05	0.027
**HADS-D**		-0.02	0.227	-0.01	0.753
**HADS-T**		-0.09	**0.014** [c)]	-0.05	0.144
	**Diagnosis** (UC/ CD)				
**HADS-A**		0.05	0.928	0.05	0.932
**HADS-D**		-0.26	0.613	-0.52	0.296
**HADS-T**		-0.21	0.830	-0.47	0.632
**HADS-A**	Age in years	-0.06	**0.002** [c)]	-0.04	0.027
**HADS-D**		-0.02	0.236	-0.01	0.597
**HADS-T**		-0.08	**0.012** [c)]	-0.05	0.118

Abbreviations: HADS = Hospital Anxiety and Depression Scale; *HADS-A* = anxiety subscale; *HADS-D* = depression subscale; *HADS-T* = summed anxiety and depression subscale; *HBI* = Harvey-Bradshaw index; *PMS* = Partial Mayo Score; *SUSI* = Stavanger University Hospital; *UC* = ulcerative colitis; *CD* = Crohn's disease; *B* = regression coefficient.

a) Age, gender, BMI, folate, kobalamin, CRP, Calprotectin.

b) Six models were developed based on two time points (0 and 3 months) and three predictors (diagnosis, HBI, and PMS), with each predictor included separately to reduce missing values, minimize multicollinearity risk, and facilitate interpretation of predictor effects; c) Significant level was determined at Bonferroni-corrected alpha: 0.05/3 = 0.017 (for three dependent variables) and marked in **bold** figures.
