# Supplementary material for: Associations Between Anxiety, Depression, and Disease Activity in Newly Diagnosed Patients with Inflammatory Bowel Disease: A Prospective Cohort Study
Source: Nurs Rep. 2026 Aug 26;16(9):301. doi: 10.3390/nursrep16090301 (PMC13610852; doi:10.3390/nursrep16090301)
Supplement: Supplementary file 1 [file nursrep-16-00301-s001.zip › Supplementary Table S6.pdf]

Supplementary Table S6. Differences between UC and CD in Dichotomized HADS Prevalence Scores Among Patients with Ulcerative Colitis and Crohn's Disease at Baseline and Three Months Follow-Up in the Norwegian SUSI Study (2012-2019) Involving 304 Patients with IBD.

Dichotomized HADS-scores	Month	Diagnosis	N	n	%	Mean	SD	95% CI	Difference between UC/CD p-value
**Anxiety (HADS-A ≥ 8)**	0	UC	225	69	30.7	5.78	4.03	(0.25,0.37)	
	0	CD	77	21	27.3	5.69	3.50	(0.17,0.37)	
									0.665
	3	UC	224	44	19.6	4.86	3.65	(0.14,0.25)	
	3	CD	77	21	27.3	5.48	3.73	(0.17,0.37)	
									0.198
**Depression (HADS-D ≥ 8)**	0	UC	225	31	13.8	3.37	3.40	(0.09,0.18)	
	0	CD	77	12	15.6	3.61	3.39	(0.07,0.24)	
									0.707
	3	UC	224	20	8.9	2.67	3.08	(0.05,0.13)	
	3	CD	77	12	15.6	3.43	3.60	(0.07,0.24)	
									0.132
**Combined anxiety/depression (HADS-T ≥ 15)**	0	UC	225	52	23.1	9.15	6.72	(0.18,0.29)	
	0	CD	77	15	19.5	9.30	6.27	(0.10,0.29)	
									0.531
	3	UC	224	34	15.2	7.54	6.20	(0.10,0.20)	
	3	CD	77	15	19.5	8.91	6.64	(0.10,0.29)	
									0.474

Abbreviations: *SUSI* = Stavanger University Hospital IBD study; *IBD* = Inflammatory Bowel Disease; *HADS* = Hospital Anxiety and Depression Scale; *HADS-A* = anxiety subscale; *HADS-D* = depression subscale; *HADS-T* = anxiety + depression subscale summarized.

Note; Although the colored percentages are the same, the corresponding means and standard deviations are different. However, the differences are small, and when the cutoff value is set at 8, 8, and 15 for defining anxiety, depression and combined anxiety/depression respectively, it will be almost the same individuals who exceed the thresholds at 0 and 3 months in the CD group.
