# Supplementary material for: Associations Between Anxiety, Depression, and Disease Activity in Newly Diagnosed Patients with Inflammatory Bowel Disease: A Prospective Cohort Study
Source: Nurs Rep. 2026 Aug 26;16(9):301. doi: 10.3390/nursrep16090301 (PMC13610852; doi:10.3390/nursrep16090301)
Supplement: Supplementary file 1 [file nursrep-16-00301-s001.zip › Supplementary Table S5.pdf]

Supplementary Table S5. Comparison of HADS Scores at Baseline and Three Months Post-Treatment Between Patients with Ulcerative Colitis (UC) and Crohn's Disease (CD) for 304 Patients Included in the Norwegian SUSI Study (2012-2019).

		UC	CD			t-test	MW	Effect size
**HADS scales**	**m**	**Mean (SD)**	**Mean (SD)**	**Mean difference**	**95% CI**	**p**	**p**	**d**
HADS-A	0	5.78 (4.03)	5.69 (3.50)	0.09	(-0.92, 1.11)	0.856	0.871	0.02
	3	4.86 (3.65)	5.48 (3.73)	-0.62	(-1.57, 0.34)	0.203	0.204	-0.17
HADS-D	0	3.37 (3.40)	3.61 (3.39)	-0.24	(-1.12, 0.64)	0.590	0.538	-0.07
	3	2.67 (3.08)	3.43 (3.60)	-0.75	(-1.59, 0.08)	0.077	0.131	-0.23
HADS-T	0	9.15 (6.72)	9.30 (6.27)	-0.15	(-1.86, 1.57)	0.866	0.627	-0.02
	3	7.54 (6.20)	8.91 (6.64)	-1.37	(-3.01, 0.27)	0.101	0.082	-0.22

Abbreviations: SUSI = Stavanger University Hospital IBD Study; *HADS* = Hospital Anxiety and Depression Scale; *HADS-A* = Anxiety Subscale; *HADS-D* = Depression Subscale; *HADS-T* = Combined Anxiety and Depression Subscale; *m* = months; *SD* = Standard Deviation; *CI* = Confidence Interval; *t* = Unpaired t-test; *MW* = Mann-Whitney Test; *P* = Two-sided p-value; *d* = Cohen's d

Følsomhet Intern (gul)
