# Supplementary material for: Associations Between Anxiety, Depression, and Disease Activity in Newly Diagnosed Patients with Inflammatory Bowel Disease: A Prospective Cohort Study
Source: Nurs Rep. 2026 Aug 26;16(9):301. doi: 10.3390/nursrep16090301 (PMC13610852; doi:10.3390/nursrep16090301)
Supplement: Supplementary file 1 [file nursrep-16-00301-s001.zip › Supplementary Table S4.pdf]

Supplementary Table S4. Comparison of HADS Scores at Baseline and Three Months Post-Treatment Between Males and Females for 304 Patients with IBD Included in the Norwegian SUSI Study (2012-2019).

		**Males**	**Females**	**Difference**		**t-test**	**MW**	**Effect size**
**HADS scales**	**m**	**Mean (SD)**	**Mean (SD)**	**Mean**	**95% CI**	**p**	**p**	**Cohen's d**
HADS-A	0	5.32 (3.8)	6.27 (4.0)	-0.95	(-1.83, -0.07)	**0.034**	**0.032**	-0.21
	3	4.55 (3.5)	5.57 (3.8)	-1.02	(-1.85, -0.19)	**0.016**	**< 0.001**	-0.28
HADS-D	0	3.28 (3.4)	3.61 (3.4)	-0.33	(-1.11, 0.44)	0.392	0.329	-0.10
	3	2.72 (3.2)	3.04 (3.3)	-0.33	(-1.06, 0.41)	0.382	0.328	-0.10
HADS-T	0	8.6 (6.4)	9.88 (6.8)	-1.29	(-2.78, 0.20)	0.090	0.096	-0.20
	3	7.27 (6.1)	8.61 (6.5)	-0.33	(-2.78, 0.09)	0.066	**0.046**	-0.21

Abbreviations: SUSI = Stavanger University Hospital IBD Study; *IBD* = Inflammatory Bowel Disease; *HADS* = Hospital Anxiety and Depression Scale; *HADS-A* = Anxiety Subscale; *HADS-D* = Depression Subscale; *HADS-T* = Combined Anxiety and Depression Subscale; *m* = months; *SD* = Standard Deviation; *CI* = Confidence Interval; *t* = Unpaired t-test; *MW* = Mann-Whitney Test; *P* = Two-sided p-value (**Bold** = $p < 0.05$).

Følsomhet Intern (gul)
