# Supplementary material for: Associations Between Anxiety, Depression, and Disease Activity in Newly Diagnosed Patients with Inflammatory Bowel Disease: A Prospective Cohort Study
Source: Nurs Rep. 2026 Aug 26;16(9):301. doi: 10.3390/nursrep16090301 (PMC13610852; doi:10.3390/nursrep16090301)
Supplement: Supplementary file 1 [file nursrep-16-00301-s001.zip › Supplementary Table S3.pdf]

Supplementary Table S3. Prevalence and Impact of Clinical Remission on Anxiety and Depression from Baseline to Three Months for 304 Patients with Ulcerative Colitis or Crohn's Disease Included in the Norwegian SUSI Study (2012-2019).

**Disease type**	**Time months**	**Clinical remission n (%)**	**Anxiety association χ_1^2 (p-value)**	**Depression association χ_1^2 (p-value)**
**Ulcerative colitis**	0	43 (19.3)	2.57 (0.109)	0.24 (0.623)
	3	177 (78.0)	6.42 **(0.011)**	9.90 **(0.002)**
**Crohn´s disease**	0	43 (54.0)	4.40 **(0.036)**	7.07 **(0.008)**
	3	59 (78.7)	9.93 **(0.002)**	3.52 (0.061)

Abbreviations: SUSI = Stavanger University Hospital IBD Study; *Clinical Remission* = Partial Mayo Score ≤ 2 and Harvey-Bradshaw Index < 5; *Anxiety* = Hospital Anxiety and Depression Scale, Anxiety Subscale ≥ 8; *Depression* = Hospital Anxiety and Depression Scale, Depression Subscale ≥ 8; χ_1^2 = Pearson's Chi-Square Value with 1 degree of freedom. (**Bold** = p ≤ 0.05).
