# Supplementary material for: Associations Between Anxiety, Depression, and Disease Activity in Newly Diagnosed Patients with Inflammatory Bowel Disease: A Prospective Cohort Study
Source: Nurs Rep. 2026 Aug 26;16(9):301. doi: 10.3390/nursrep16090301 (PMC13610852; doi:10.3390/nursrep16090301)
Supplement: Supplementary file 1 [file nursrep-16-00301-s001.zip › Supplementary Table S2.pdf]

Supplementary Table S2. Correlation Between HADS Scores and Disease Indicators, Biomarkers, and Body Mass Index (BMI) at Baseline and Three Months Post-Treatment for 304 Patients with IBD Included in the Norwegian SUSI Study (2012-2019).

	**Time**			**HADS-A**		**HADS-D**		**HADS-T**	
**Disease indicators**	**months**	**Mean**	**SD**	**r**	**p**	**r**	**p**	**r**	**p**
HBI	0	5.34	3.61	0.41	**< 0.001**	0.36	**0.001**	0.42	**< 0.001**
	3	3.56	2.80	0.42	**< 0.001**	0.37	**< 0.001**	0.44	**< 0.001**
PMS	0	4.98	2.43	0.15	**0.022**	0.14	**0.033**	0.17	**0.014**
	3	1.46	1.90	0.17	**0.010**	0.26	**< 0.001**	0.23	**< 0.001**
CRP, *mg/L*	0	19.3	30.7	0.04	0.535	0.14	**0.017**	0.10	0.079
	3	6.2	11.4	0.10	0.101	0.13	**0.024**	0.12	**0.036**
Calprotectin, *mg/kg*	0	1111.2	1122.2	-0.02	0.719	0.07	0.277	0.02	0.723
	3	341.4	650.1	0.01	0.871	0.07	0.301	0.04	0.536
**Biomarkers + Body Mass Index**									
Folate, *nmol/L*	0	14.1	8.0	0.02	0.753	-0.03	0.557	-0.01	0.906
	3	17.0	8.7	0.09	0.121	0.08	0.171	0.09	0.110
Cobalamin, *pmol/L*	0	426.1	234.3	0.03	0.562	0.01	0.912	0.02	0.690
	3	373.4	184.2	-0.07	0.232	0.02	0.732	-0.03	0.607
BMI, *kg/m²*	0	24.6	4.6	-0.05	0.375	-0.00	0.966	-0.03	0.587
	3	24.8	4.3	-0.04	0.496	0.06	0.303	0.01	0.895

Abbreviations: SUSI = Stavanger University Hospital IBD Study; *IBD* = Inflammatory Bowel Disease; *HADS* = Hospital Anxiety and Depression Scale; *HADS-A* = Anxiety Subscale; *HADS-D* = Depression Subscale; *HADS-T* = Combined Anxiety and Depression Subscale; *HBI* = Harvey-Bradshaw Index (Scale 0-25); *PMS* = Partial Mayo Score (Scale 0-9); *CRP* = C-Reactive Protein; *Calprotectin* = Fecal Calprotectin; *BMI* = Body Mass Index; *r* = Pearson Product-Moment Correlation Coefficient; *SD* = Standard Deviation. **Reference Ranges:** CRP (<5), Folate (>6.8), BMI (18.5-24.9), Cobalamin cutoff for disease indication (>250); Calprotectin: Normal (<50); Cutoff for disease indication (>250) (**Bold** = $p < 0.05$).
