# Supplementary material for: Associations Between Anxiety, Depression, and Disease Activity in Newly Diagnosed Patients with Inflammatory Bowel Disease: A Prospective Cohort Study
Source: Nurs Rep. 2026 Aug 26;16(9):301. doi: 10.3390/nursrep16090301 (PMC13610852; doi:10.3390/nursrep16090301)
Supplement: Supplementary file 1 [file nursrep-16-00301-s001.zip › Supplementary Table S1.pdf]

Supplementary Table S1. Changes in Dichotomized HADS Scores from Baseline to Three Months Post-Treatment in 304 Patients with IBD Included in the Norwegian SUSI Study (2012-2019).

	**Time**	**Descriptives**		**McNemar's**
**Dichotomized HADS-scores for IBD**	**months**	**n**	**%**	**p-value**
Anxiety (HADS-A ≥ 8)	0	90	29.8	
	3	65	21.6	**< 0.001**
Depression (HADS-D ≥ 8)	0	43	14.2	
	3	32	10.6	0.117
Combined anxiety/depression (HADS-T ≥ 15)	0	67	22.2	
	3	49	16.3	**0.003**

Abbreviations: SUSI = Stavanger University Hospital IBD Study; *IBD* = Inflammatory Bowel Disease; *HADS* = Hospital Anxiety and Depression Scale; *HADS-A* = Anxiety Subscale; *HADS-D* = Depression Subscale; *HADS-T* = Combined Anxiety and Depression Subscale. (**Bold** = $p < 0.05$).
